# Protective antigenic epitopes revealed by immunosignatures after three doses of inactivated SARS-CoV-2 vaccine

**DOI:** 10.3389/fimmu.2022.938378

**Published:** 2022-08-09

**Authors:** Mian Peng, Xiaowen Dou, Xiuming Zhang, Mingchen Yan, Dan Xiong, Ruiwei Jiang, Tong Ou, Aifa Tang, Xiqiu Yu, Feiqi Zhu, Weiqin Li

**Affiliations:** ^1^ The First School of Clinical Medicine, Southern Medical University, Guangzhou, China; ^2^ Department of Critical Care Medicine, Affiliated Jinling Hospital, Medical School of Nanjing University, Nanjing, China; ^3^ Department of Critical Care Medicine, The Third Affiliated Hospital of Shenzhen University, Shenzhen, China; ^4^ Medical Laboratory, The Third Affiliated Hospital of Shenzhen University, Shenzhen, China; ^5^ Department of Artificial Intelligence and Bioinformatics, Shenzhen Digital Life Research Institute, Shenzhen, China; ^6^ Science and Education Center, The Third Affiliated Hospital of Shenzhen University, Shenzhen, China; ^7^ Department of Gastroenterology, The Third Affiliated Hospital of Shenzhen University, Shenzhen, China; ^8^ Department of Neurology, The Third Affiliated Hospital of Shenzhen University, Shenzhen, China

**Keywords:** protective antigenic epitopes, immunosignatures, three doses, inactivated, SARS-CoV-2 vaccine

## Abstract

**Background:**

SARS-CoV-2 (severe acute respiratory syndrome coronavirus 2) has infected millions of people around the world. Vaccination is a pillar in the strategy to control transmission of the SARS-CoV-2 spread. Immune responses to vaccination require elucidation.

**Methods:**

The immune responses to vaccination with three doses of inactivated SARS-CoV-2 vaccine were followed in a cohort of 37 healthy adults (18–59 years old). Blood samples were collected at multiple time points and submitted to peptide array, machine learning modeling, and sequence alignment analyses, the results of which were used to generate vaccine-induced antibody-binding region (VIABR) immunosignatures (Registration number: ChiCTR2200058571).

**Results:**

Antibody spectrum signals showed vaccination stimulated antibody production. Sequence alignment analyses revealed that a third vaccine dose generated a new highly represented VIABR near the A570D mutation, and the whole process of inoculation enhanced the VIABR near the N501Y mutation. In addition, the antigen conformational epitopes varied between short- and long-term samples. The amino acids with the highest scores in the short-term samples were distributed primarily in the receptor binding domain (RBD) and N-terminal domain regions of spike (S) protein, while in the long-term samples (12 weeks after the 2^nd^ dose), some new conformational epitopes (CEs) were localized to crevices within the head of the S protein trimer.

**Conclusion:**

Protective antigenic epitopes were revealed by immunosignatures after three doses of inactivated SARS-CoV-2 vaccine inoculation. A third dose results in a new top-10 VIABR near the A570D mutation site of S protein, and the whole process of inoculation enhanced the VIABR near the N501Y mutation, thus potentially providing protection from strains that have gained invasion and immune escape abilities through these mutation.

## Background

SARS-CoV-2 (severe acute respiratory syndrome coronavirus 2) is the highly pathogenic virus that caused the COVID-19 (coronavirus 2019) pandemic following its emergence in late 2019. Its emergence followed two other newly identified highly pathogenic coronaviruses, namely those that caused the Middle East respiratory syndrome and original SARS outbreaks in the late 20^th^ and early 21^st^ centuries, respectively (1). An effective vaccine is considered to be essential for limiting SARS-CoV-2 transmissibility and for protecting against severe COVID-19 morbidity and mortality (2). Most of the current COVID-19 vaccine research focuses on virus-vectored vaccines or mRNA vaccines (3–6). So far, there have been limited studies on inactivated SARS-CoV-2 vaccines.

The efficacy of Sinovac’s inactivated SARS-CoV-2 vaccine (ISC2V) is being examined in an ongoing clinical trial of healthcare professionals with the incidence of symptomatic virologically confirmed COVID-19 cases two or more weeks after the second vaccination serving as the primary efficacy endpoint. Results from this trial have not yet been published (7). A prior report of the findings of phase 1 and phase 2 ISC2V trials conducted in China showed low adverse reaction rates and demonstrated immunogenicity, leaving questions regarding efficacy and possible long-term adverse events for phase 3 trials (8). The epitopes that antibodies generate in response to inactivated SARS-CoV-2 vaccine target have not yet been reported. The aims of the present study were firstly to examine the immune efficacy of ISC2V in terms of its ability to induce human immune spectrum changes and secondly to identify the antigenic epitopes of vaccine action by way of immunosignatures developed based on peptide array technology.

## Methods

### Participants

A cohort of 37 healthy adults who agreed to receive three ISC2V doses were recruited into the study. The inclusion criteria were: being 18–59 years old; having negative nucleic acid detection and specific IgM, IgG SARS-CoV-2 tests; being in good health without any known underlying diseases; ISC2V acceptance; and not having a serious adverse reaction during vaccination with ISC2V (9). The exclusion criteria beyond not meeting the aforementioned inclusion criteria were: being pregnant or becoming pregnant during follow-up (including miscarriage/abortion); suffering an acute illness requiring hospitalization during follow-up; and choosing to withdraw from the study for any reason. Written informed consent was obtained from each participant prior to commencement of the study. Ethics approval was obtained from the Ethics Committee of the Third Affiliated Hospital of Shenzhen University.

### Experimental timepoints

The data were analyzed over nine time points, with each time point being defined relative to the most recent prior vaccine injection, including whether it was the first, second, or third vaccine dose (indicated as Injection I, II, or III) and how many weeks (indicated as 2w, 4w, 12w, 24w, or 32w) have passed since that most recent injection. Thus, the nine study time points were: Injection I-2w; Injection I-4w; Injection II-2w; Injection II-4w; Injection II-12w; Injection II-24w; Injection II-32w; Injection III-2w; and Injection III-4w. Naïve immunity baseline data for comparison are referred to as Injection I-0 data. Additionally, the Injection I-4w and Injection II-32w time points served as baseline reference data for Injection II and Injection III, respectively, at which times they are referred to as Injection II-0 and Injection III-0, respectively.

### Descriptive data

The following data were collected for each participant: gender, age, height (cm), body weight (kg), disease history, ISC2V injection dates, and any local or systemic adverse reactions after each injection.

### Biological sample preparation

At each time point, 1 ml of whole blood was drawn from each subject and centrifuged at 3500 RPM for 5 min. The upper serum fraction was taken and stored at -80°C. Sample aliquots were thawed at 4°C and 3 μl of each sample was diluted 1:25 in 72 μl mannitol buffer [1% mannitol in PBSTP (phosphate buffered saline with 0.05% Tween20 and 0.1% ProClin™); ingredients from Sigma-Aldrich, USA]. Then, 5-μl aliquots of the 1:25 sample dilutions were transferred into the 96-well plates (Axygen Scientific, Union City, CA), where they were combined with 120 μl of PBSTP to produce 1:625 dilutions for use in the assay.

### Peptide assays

Peptide arrays were rehydrated by soaking in distilled water for 20 min. Rehydrated arrays were sprayed three times briefly with 90% isopropanol (Thermo Fisher Scientific, USA) ensuring full coverage, centrifuged to remove excess liquid, and then loaded into a peptide array cassette. Each sample was diluted 1:625 in mannitol buffer (1% mannitol in PBSTP), and then 90 μl of the diluted sample was transferred to the cassette. To facilitate antibody-peptide binding, the mixture was incubated on the arrays for 1 h at 37°C while shaking on a TeleShake95 shaker (INHECO, Martinsried, Germany). Subsequently, the cassette was washed 10 times in PBSTP with a BioTek 405TS microplate washer (BioTek Instruments, Winooski, VT, USA). Bound antibody was detected by adding 4 nM goat anti-human IgG (H+L) secondary antibody coupled to AlexaFluor 555 (Thermo Fisher Scientific, USA) in secondary incubation buffer [0.75% casein (Sigma-Aldrich, USA) in PBSTP] for 1 h at 37°C shaking on a TeleShake95 shaker. After incubation with the secondary antibody, the peptide arrays were again washed with PBSTP, followed by a rinse in distilled water. After being removed from the cassette, the peptide arrays were sprayed with 90% isopropanol and excess liquid was removed by centrifugation at 3000 RPM for 60 s.

### Data acquisition and processing

Peptide arrays were scanned individually in an ImageXpress Micro 4 imaging system (Molecular Devices, Santa Clara, CA), which generated TIFF images. The images were analyzed in MIAMI software wherein an automated grid algorithm was applied to identify areas associated with each peptide, yielding a GPR5 format data matrix. The original data foreground (FG) of each polypeptide was extracted from the data matrix in GPR5 format. Because the original fluorescence signal data show a lognormal distribution, log-FG was obtained by logarithmic transformation after adding the constant 100 to the FG to improve homovariance. Data measurement accuracy was roughly proportional to intensity. The median of all peptides in each array was subtracted from the peptide signal log-FG to obtain a normalized log-FG.

### Difference analysis of peptide signal

At each sampling time point, each peptide signal (features of normalized log-FG) was ranked according to its *p* value in a *t*-test relative to baseline (pre-vaccination) levels. A 5% false discovery rate (FDR) threshold that distinguished clearly between before and after vaccination states was applied and those peptides with a significant difference whose fold-change of the original signal was greater than 1 after vaccination were identified as significantly differentiated peptides (SDPs). If the number of peptides reaching the threshold was less than 100, then the top 1000 peptides with the lowest *p* values were selected as SDPs.

### Polypeptide sequence alignment scoring

To reveal potential epitopes of vaccine action, SDP sequences were aligned with Spike (S) protein sequences obtained from a SARS-CoV-2 reference proteome (National Center for Biotechnology Information, NC_045512) (10) *via* Basic Local Alignment Search Tool (BLAST) analysis (seed 3, gap penalty 4), and the scoring matrix BLOSUM62 was adjusted according to the amino acid composition of the peptide arrays (11). Each reference sequence area was aligned with a group of SDPs. The sum of all aligned SDPs within an area served as an amino acid score, a. To correct for array composition deviation, we use the same method to calculate the background score, b, of all peptide arrays in the location. The number of SDPs screened out was recorded as c, and the number of all peptide arrays was recorded as d. Each final score, s, was calculated as follows: s = a – (c/d) × b. Total scores of 20-mer sequence spans were obtained by window sliding. The top-10 total scores for non-overlapping S protein sequence spans were accepted as linear vaccine-induced antibody-binding regions (VIABRs).

### Patch-based conformational epitope identification

After obtaining the complete spatial structure file for S Protein from https://charmm-gui.org (12), the Dictionary of Secondary Structure of Protein (13, 14) was used to analyze S Protein Structure and to calculate the relative surface accessibility of each residue. Residues on the protein surface were obtained according to the residual relative surface accessibility, and overlapping residue-centered patches were generated. SDPs were aligned with the patches based on maximal bipartite matching in a bipartite graph, and alignment scores were calculated with the aa similarity matrix (15–17). Post-processing (clustering, merges, and gap filling) were carried out according to alignment scoring to obtain candidate CE information. Visualization of CEs relative to protein spatial structure was conducted in Pymol software.

### Consistency analysis with SARS-CoV-2 neutralizing antibodies

The amino acid composition of each CE was compared with that extracted from the binding site of each SARS-CoV-2 neutralizing antibody (downloaded from the immune epitope database). When there was >80% amino acid identity, a sequence was considered to match a neutralizing antibody CE. The identity of each thus matched CE was recorded.

### Scoring model

Because machine learning modeling requires sample training to follow the principle of independent and identical distribution, we set up antibody level scoring models separately for short-term samples and long-term samples. In the short-term sample scoring model, sample pools within 4 weeks after each inoculation (Injection I-2w; Injection I-4w; Injection II-2w; Injection II-4w; Injection III-2w; and Injection III-4w) were used as training-set case samples. In the long-term sample scoring model, sample pools beyond 4 weeks (Injection II-12w; Injection II-24w; and Injection II-32w) were used as training-set case samples. The Injection I-0 (prevaccination) sample was used as a baseline control for both models. Dichotomous predictions were performed, and the predicted probabilities were used as antibody level scores. A Venn diagram was made to identify which peptide signals were shared and which were not shared between long-term and short-term samples. The leave-one-out method, enhanced to adjust to queue characteristics, was used to prevent information leakage consequent to the donor data distributions of the training and test sets. Logistic regression was used to construct the classification model. Receiver operating characteristic (ROC) curves were produced for each model and the area under the curve (AUC) was determined for each ROC curve.

### Statistical analysis

Data processing and hypothesis testing were performed in R3.6 and Python3.7. Differences in model prediction scores between samples of different sampling points were calculated with *t*-tests. Differences with a *P* value <*0.05* were considered significant.

## Results

### Baseline characteristics of the participants

A total of 37 participants (23 women and 14 men) were enrolled. They had a median ( ± standard deviation) age of 32.51 ± 6.50 years and body mass index (BMI) of 21.71 ± 3.24 kg/m^2^, respectively. The age, height, weight, and BMI for each participant are shown in **Table 1**. All 37 participants followed a three-injection ISC2V inoculation protocol with a 4-week interval from the first to the second injection and a 32-week interval from the second to the third injection. None of the participants had preexisting diseases or severe adverse reactions to ISC2V inoculation.

**Table 1:** Baseline data for each participant.

	Gender	Age(years)	Height(cm)	Weight(kg)	BMI(kg/m^2^)
**C1**	Female	31	163	50	18.82
**C2**	Female	36	164	54	20.08
**C3**	Female	28	164	50	18.59
**C4**	Female	24	163	47	17.69
**C5**	Female	32	159	52	20.57
**C6**	Female	27	157	50	20.28
**C7**	Male	31	173	65	21.72
**C8**	Female	26	157	50	20.28
**C9**	Female	32	160	51	19.92
**C10**	Female	27	157	50	20.28
**C11**	Male	36	173	65	21.72
**C12**	Male	31	172	60	20.28
**C13**	Male	32	169	60	21.01
**C14**	Male	45	175	70	22.86
**C15**	Female	28	162	55	20.96
**C16**	Female	30	158	50	20.03
**C17**	Female	55	160	47	18.36
**C18**	Male	30	172	78	26.37
**C19**	Male	29	187	105	30.03
**C20**	Female	27	165	55	20.20
**C21**	Male	48	170	65	22.49
**C22**	Female	33	163	60	22.58
**C23**	Female	38	155	60	24.97
**C24**	Female	32	161	50	19.29
**C25**	Female	33	153	48.5	20.72
**C26**	Female	43	160	60	23.44
**C27**	Female	33	158	55	22.03
**C28**	Female	27	155	60	24.97
**C29**	Male	26	160	70	27.34
**C30**	Male	33	183	75	22.30
**C31**	Male	27	174	65	21.47
**C32**	Female	28	165	50	18.37
**C33**	Male	32	174	100	33.03
**C34**	Female	30	162	50	19.05
**C35**	Male	31	168	55	19.49
**C36**	Male	39	170	60	20.76
**C37**	Female	33	155	50	20.81

### Vaccine antibody response-associated polypeptides

**Figure 1:**
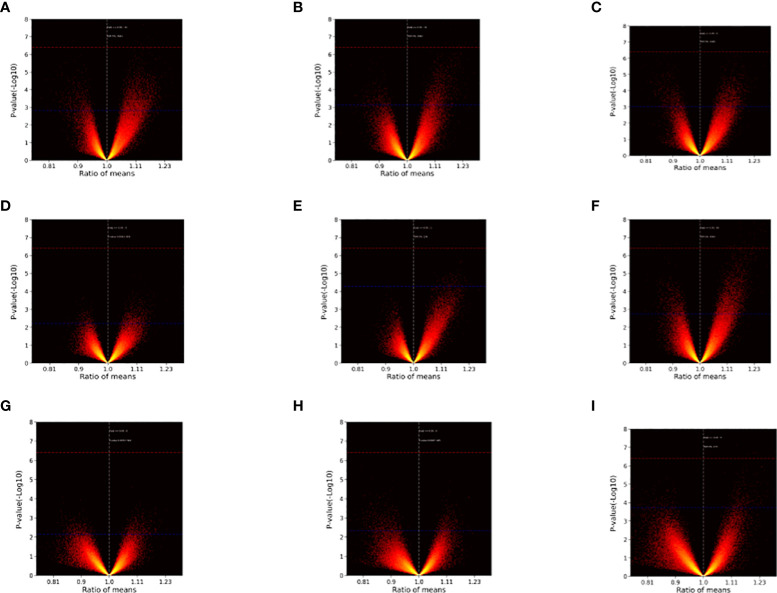
Volcano plots illustrating inter-group differences. **(A)** Injection I-2w vs. Injection I-0. **(B)** Injection I-4w vs. Injection I-0. **(C)** Injection II-2w vs. Injection I-0. **(D)** Injection II-4w vs. Injection I-0. **(E)** Injection II-12w vs. Injection I-0. **(F)** Injection II-24w vs. Injection I-0. **(G)** Injection II-32w vs. Injection I-0. **(H)** Injection III-2w vs. Injection I-0. **(I)** Injection III-4w vs. Injection I-0. The ratio of means for each peptide array post-inoculation across different time points relative to the baseline are shown on the x-axes. The significance of the comparisons [p value (-Log 10)] is represented on the y-axes. Red dashed lines indicate Bonferroni-corrected p = 0.05 threshold. Blue dashed lines indicate 5% FDR boundary. Note that ratios with greater (absolute) values tended to be more significant and differences (ratio > 1) emerged at every time point.

After sample processing and data acquisition, 100~5000 SDPs were identified at each time point. Volcano plots (**Figure 1**) were produced to illustrate the significance of differences across time points as a function of the ratio of means of each post-inoculation peptide array across the experimental time points relative to the pre-inoculation baseline. Notably, SDPs with ratio values greater than 1 were observed for every inoculation-timepoint group and larger ratios tended to have more statistically significant differences, suggesting that inoculation can promote the production of effector antibodies.

### Sequence alignment

**Figure 2:**
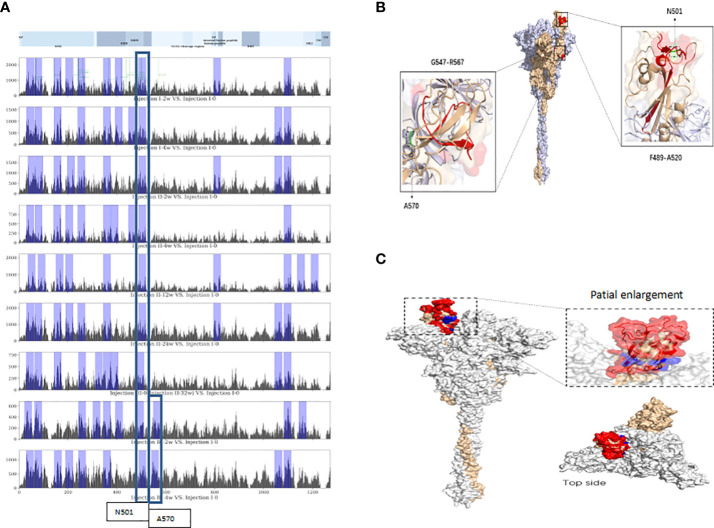
Linear and conformational epitopes identified following sequence alignment. **(A)** Top-10 scoring sequence spans in protein S (non-overlapping) at each assessment time point located near N501 or A570. **(B)** Three-dimensional structure positions of A570 and N501. **(C)** The CEs of the RBD. Red-highlighted aa residues were consistent spatial epitopes across all timepoints, and those marked in blue are located within the crevices of the spatial structure of the head of the S-protein trimer and were observed in long-term sample analyses (12 weeks after the 2nd dose).

Following alignment of vaccine-induced antibody response-associated SDPs at each sampling time point with reference-proteome SARS-CoV-2 S protein sequences, the top-10 total scores for non-overlapping 20-mer amino acid-sequence spans from the S protein were identified as linear VIABRs (highlighted in **Figure 2A**). The three-dimensional structure positions of A570 and N501 are shown in **Figure 2B**.

Following alignment of the spatial structure of SDPs with those of the SARS-CoV-2 S protein, we identified 6~8 corresponding CEs at each time point. Each identified CE is composed of 12~41 aa which are discontinuous in sequence but relatively close in space. The CEs of the receptor binding domain (RBD) are shown in **Figure 2C**.

### Peptide array-based antibody level scoring

**Figure 3:**
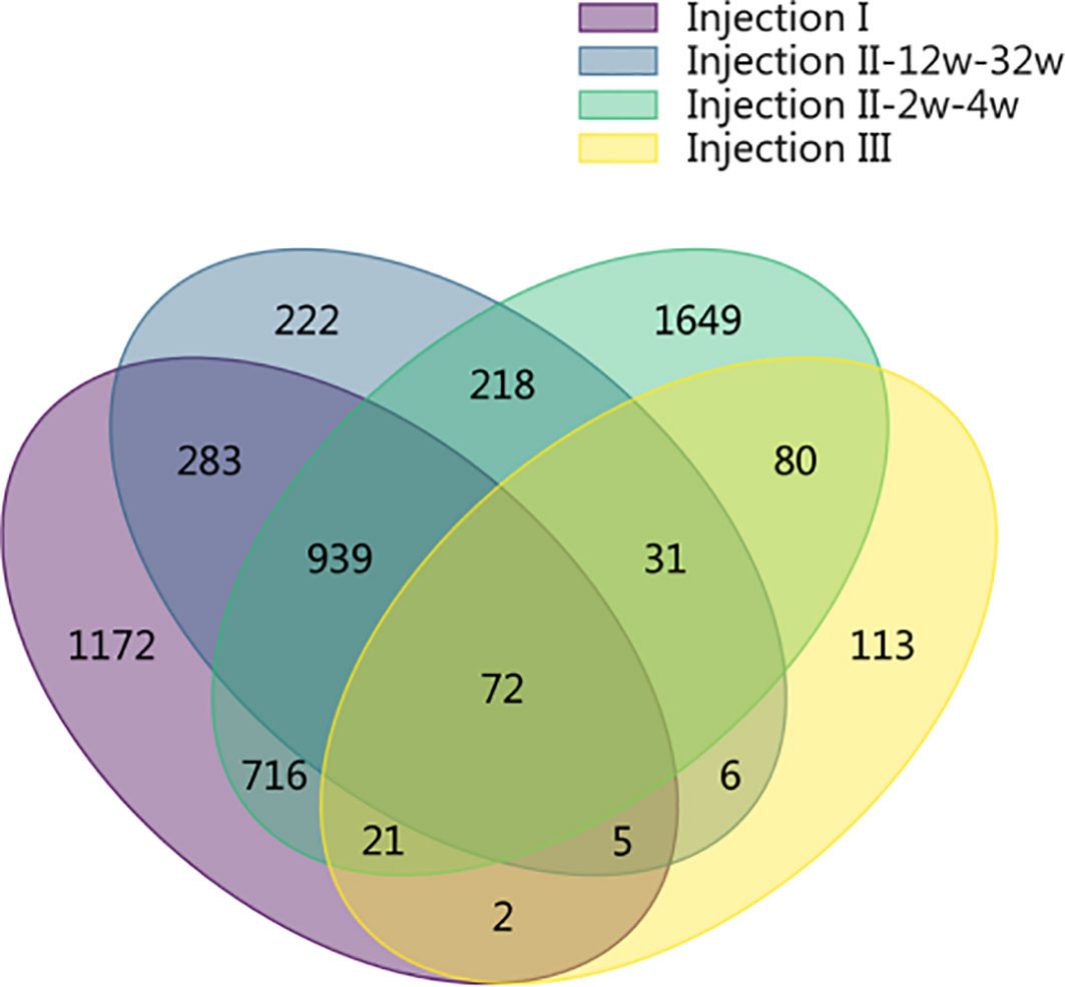
Venn diagram showing the SDPs that were unique identified to each phase of the inoculation protocol. In the post-injection I period and in the post-injection III period, we identified 1,172 and 113 unique SDPs, respectively. Altogether, there were 2,089 SDPs unique to the post-Injection II period, only 218 of which were found for both the short-term and long-term samples. Of the remaining 1,871 SDPs from this period, 222 were unique to the long-term samples while 1,649 were unique to the short-term samples (FDR = 0.05). Only 72 SDPs were common throughout the entire immunization cycle, indicating that the long- and short-term samples showed different antibody binding patterns.

The SDPs that were unique identified to each phase of the inoculation protocol are shown in a Venn Diagram in **Figure 3**. In the post-injection I period and in the post-injection III period, we identified 1,172 and 113 unique SDPs, respectively. Altogether, there were 2,089 SDPs unique to the post-Injection II period, only 218 of which were found for *both* the short-term and long-term samples. Of the remaining 1,871 SDPs from this period, 222 were unique to the long-term samples while 1,649 were unique to the short-term samples (FDR = 0.05). Only 72 SDPs were common throughout the entire immunization cycle, indicating that the long- and short-term samples showed different antibody binding patterns.

**Figure 4:**
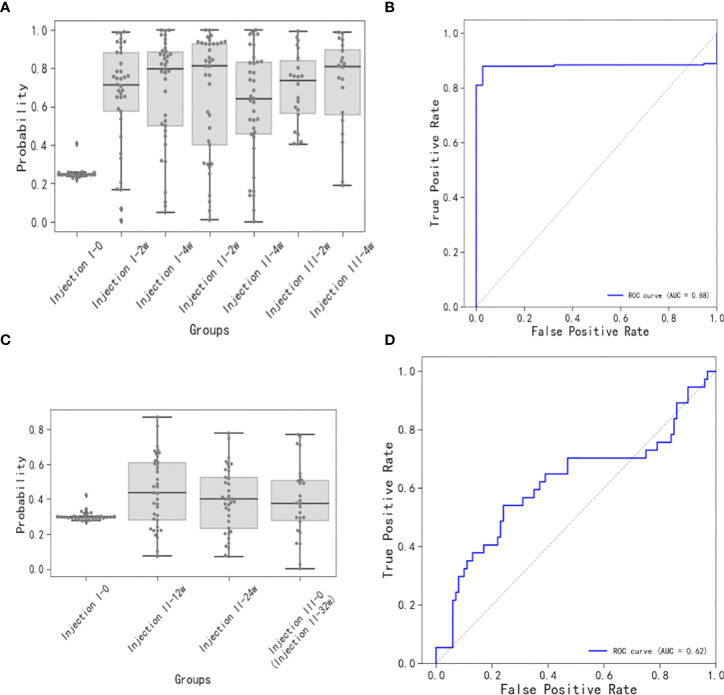
Short-term and long-term models of immune response to vaccination. **(A)** Distributions of short-term immune response scores. Note that the baseline sample score was ~0.25 and the post-inoculation scores were generally greater than 0.65, increasing gradually up to the Injection II-2w assessment, then decreasing at the Injection II-4w assessment, and increasing gradually up until the Injection III-4w assessment. **(B)** ROC for scores over time (AUC = 0.88) for the short-term model. **(C)** Distributions of long-term immune response scores. Scores at the Injection II-12w assessment were slightly higher than baseline and then proceeded to decrease thereafter. **(D)** ROC for scores over time (AUC = 0.62) for the long-term model.

The short-term sample model (**Figure 4A**), whose ROC had an AUC of 0.88 (**Figure 4B**), had a baseline sample score of ~0.25, while the post-inoculation scores exceeded 0.65. Scores increased gradually with time after the inoculation until they reached maximum levels at the Injection II-2w time points. By the Injection II-4w time point, the scores decreased to 0.65. Subsequently, scores increased gradually until the Injection III-4w time point, when they reached levels similar to that seen at Injection II-2w.

The long-term sample model (**Figure 4C**), whose ROC had an AUC of 0.62 (**Figure 4D**), showed that the score distribution at the Injection II-12w time point skewed to slightly greater values than those observed at baseline and then proceeded to decrease at subsequent time points (Injection II-24w and -32w), indicating that some antibodies were still being produced 12 weeks after inoculation, though these signals were trending toward a state of extinction.

### Classification of immune responses based on antibody level scoring of polypeptide arrays

**Figure 5:**
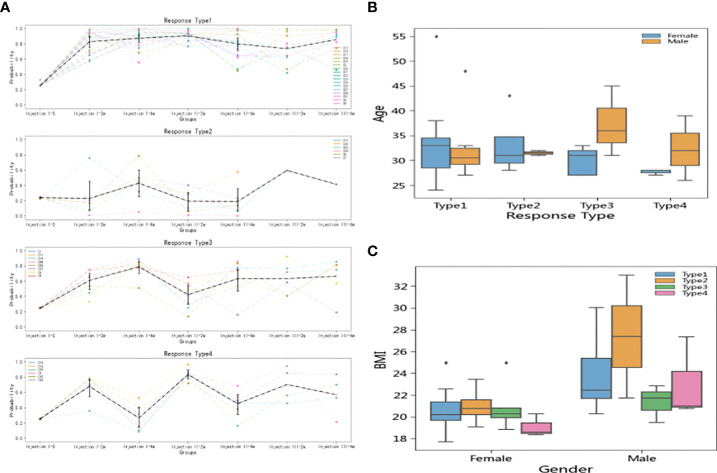
Inoculation reaction types and relationship of types to individual factors. **(A)** Types were defined according to k-means clustering, with Type 1 showing strong antibody responses, Type 2 showing weak responses until the third dose, Type 3 showing a lasting response to the first dose, and Type 4 showing a notable boosts in immune response to follow-up doses. **(B)** There was no significant difference between the age distributions of men and women in terms of vaccine response. **(C)** Type 2 participants (poor responders to doses 1 and 2) had higher BMI values than participants in the other type groups. The effect was significant in both women and men, but dramatically more pronounced in men than in women.

In the short-term model, the 37 participants could be divided into four reaction types based on k-means clustering (**Figure 5A**): Type 1 (N = 17), Type 2 (N = 6), Type 3 (N = 8), and Type 4 (N = 6). The Type 1 individuals had strong vaccine antibody responses indicative of vaccine effectiveness. The Type 2 individuals had weak antibody responses initially but then achieved high antibody level scores after the third injection. Type 3 and 4 were characterized by fluctuating responses over the inoculation course, with Type 3 individuals exhibiting a lasting response to the first injection, while Type 4 showed more robust responses to booster injections than to the first injection.

Analysis of the demographic characteristics showed that men and women had similar age distributions across the four types (**Figure 5B**). However, Type 2 (initially poor responder) individuals tended to have high BMIs, among both men and women, though the effect was more pronounced in men than in women (**Figure 5C**).

## Discussion

SARS-CoV-2, which emerged in China in late 2019, has infected millions of people around the world, achieving a much greater infection reach than previously known coronaviruses (18). As of the summer of 2022, the COVID-19 pandemic represented the sixth time the WHO had declared a global emergency following the H1N1 swine flu in 2009, Ebola (West Africa) in 2013, polio in 2014, Zika in 2016, and Ebola (Democratic Republic of Congo) in 2019 (19). Given the epidemiology of COVID-19, efficacious vaccination remains essential to minimizing COVID-19 morbidity and mortality.

The WHO recognizes seven COVID-19 vaccine strategies: inactivated virus; virus-like particle or nanoparticle; protein subunit; virus-vectored; DNA; mRNA; and live-attenuated virus (20). Among them, particularly supportive data have been reported for ChAdOx1, a virus-vectored vaccine, and BNT162b2, an mRNA vaccine. Notably, interim analyses of randomized controlled trials of ChAdOx1 in the UK, South Africa, and Brazil have affirmed an acceptable safety profile with good efficacy in adults (6). In another report in the UK, both ChAdOx1 and BNT162b2 were reported to produce anti-S protein IgG responses in large adult population samples, though the responses differed in relation to the number of doses received, age, gender, and long-term health conditions (21). A sentinel surveillance study conducted in Chile with 56,261 vaccinated adults showed that, compared to that in BNT162b2-inoculated participants, IgG seropositivity obtained after vaccination with CoronaVac, an inactivated virus vaccine, was lower and tended to decline more quickly (22). Because immune responses to different vaccine types and doses differ, and there are limited ISC2V studies, we could not have predicted the course of immunity acquisition that people would show over the course of receiving three doses of ISC2V inoculation. The presently identified SDPs provide information that may be useful for elucidating the mechanisms of ISC2V inoculation.

The conventional techniques that are used to assess immunogenicity and immune responses are analyses of neutralizing antibodies (NAbs). Previously, we have observed good concordance (>0.78) between conventional virus neutralization test and enzyme linked immunosorbent assay NAb results. Although NAb titers were found to decrease gradually after ISC2V vaccination in our prior work, the seropositive rate of NAb remained at 84% over 6 months of observation (23). Notably, although NAb assays based on competitive binding of angiotensin-converting enzyme 2 with the RBD remain the classic immunity marker, peptide arrays are measuring features of the response that are not measured in conventional neutralization assays. Studies using different techniques to evaluate vaccines provide more profound understanding of the vaccines and remain to be the focus of future work for the field.

Peptide microarray-based immunosignature technology captures epitope-binding of antibody immune spectrum signals and displays antibody diversity, thus providing broad information regarding circulating antibody repertoires. Using this technique, Arvey et al. characterized serum antibody binding to high-density peptide microarrays with samples from a diverse cohort of 1,675 subjects and found that the circulating antibody repertoires of older subjects showed particularly robust binding to thousands of di-serine peptide-containing peptides, which can be attributed to immune age (24). The sequence peptides are analyzed at a high density and their analysis yields CEs, which are key to evaluating vaccines, in addition to linear epitopes.

Given the difficulties inherent in producing properly folded proteins, peptide arrays are an alternative means of defining antibodies to CEs (25–27). In a study in which full reactivity profiles of vaccine-generated antibodies were produced, Legutki et al. found an antibody that was common to COVID-19 survivors while being absent in those who died (28). In the present study, by comparing immune spectra between vaccinated and non-vaccinated samples, we were able to identify SDPs as potentially relevant epitope sites. We were then able to identify and rank putative epitopes according to SDP enrichment and thus identify those most likely to be related to a SARS-CoV-2 antigen epitope.

Our linear sequence alignment analysis revealed that those sequence spans that aligned best with the S protein sequence are distributed predominantly in the RBD and N-terminal domain regions. These two regions have important functions related to viral invasion of host cells (29), and they have been shown to be the most common binding targets of SARS-CoV-2 neutralizing antibodies (30, 31). Notably, most escape mutations, including those in the UK variant B.1.1.7, Delta strains, and Omicron strains of SARS-CoV-2, have been reported to occur within these two regions (32–34).

The intersections of our top-10 S protein VIABRs with the key amino acid spans of known neutralizing antibodies published by La Jolla Institute for Immunology (LJI) (35) may be used as references for the design of polypeptide or optimized-subunit vaccines. After the third ISC2V injection, a new top-10 VIABR intersecting with a region published by LJI emerged. Near that VIABR, there is an A570D mutation, which has been shown to reduce contact between individual chains of the trimeric S protein protomer, potentially enhancing cleavage into S1 and S2 subunits, dynamic structural rearrangement, and host cell fusion mechanisms in the UK variant B.1.1.7 (36–39). The epitope region near A570 is located in a loop at beta-sheet junction (see three-dimensional structure of A570 shown in **Figure 2B**). An A570 mutation that changes antibody binding with this local structure may underlie the variation in UK Variant B.1.1.7. The fact that this VIABR is located entirely within the beta sheet suggests that a vaccine-enhanced VIABR may be protective against the UK Variant B.1.1.7.

In addition, we found a top-10 VIABR in the N501Y mutation region that persisted throughout the inoculation course of three ISC2V injections. This particular mutation has been identified as being pathogenically important in a number of strains due to its producing enhanced virus invasion and immune escape (40–42). This finding suggests that ISC2V may provide antibody protection against Delta and Omicron variants. Additionally, these data indicate that a three-dose ISC2V strategy may provide highly comprehensive protection, including against novel SARS-CoV-2 strains.

In the result, we documented distinct short- versus long-term immune response patterns following ISC2V injections. In the short term, vaccinated participants showed antibody responses that peaked about 2 weeks after the second injection and about 4 weeks after the third injection. In the long term, antibody levels showed slight increases 12 weeks after the second dose and then decreased gradually from then onward. Interestingly, antigen epitopes varied between short- and long-term samples. Our alignment CE analysis showed that the highest-scoring amino acid spans in the short-term samples were distributed primarily in the RBD and NTD regions of the S protein. Meanwhile, in long-term samples (12 weeks after the 2^nd^ dose), some new CEs were identified within the crevices of the spatial structure of the head of the S protein trimer. Thus, the long-term immunogenic effects of ISC2V may contribute to the generation of antibodies that bind to the gaps of S-protein-trimer RBD. These antibody binding pattern differences between short-term and long-term samples suggest that there is a strong short-term RBD antibody response that diminishes over time, perhaps limited by the B cell lifespan (43–45), while long-lived plasma cells produce multiple antibodies with different binding modes (46, 47). Our polypeptide array experiments also showed that vaccination can stimulate antibody production in both the short term (within 4 weeks) and the long term (beyond 4 weeks), which is consistent with prior findings obtained by Xia et al. with the inactivated SARS-CoV-2 vaccine BBIBP-CORV 27 (48), wherein humoral responses against SARS-CoV-2 were observable in all examined vaccine recipients 42 days after inoculation.

Our follow-up analyses of immune response types based on short-term model scores indicated that high-BMI individuals, especially men, were most likely to have a weak antibody production response to the vaccine (Type 2 short-term model outcome). Prior studies have provided evidence of a linear dose-response association of BMI with COVID-19 severity and mortality. Likewise, obesity per se (BMI ≥ 30 kg/m^2^) has been shown to be a risk factor for critical COVID-19 and in-hospital mortality due to COVID-19 (49–51). Although we did not observe a statistically significant correlation between BMI and vaccine efficacy in our study, it would be of interest to further determine whether obese individuals would benefit from augmented vaccine exposure relative to the general population.

This article has a notable limitation in that only 37 individuals were represented in the results. A larger population of participants will be required to further confirm the present findings and conclusions.

## Conclusion

ISC2V inoculation stimulates antibody production. After the third dose, a new top-10 VIABR was generated near the A570D mutation site of the viral S protein. A VIABR near the N501Y mutation was enhanced throughout the three-injection inoculation course. Three doses of ISC2V may provide more comprehensive antibody protection against novel variants of SARS-CoV-2 than is obtained with only two doses.

## Data availability statement

The raw data supporting the conclusions of this article will be made available by the authors, without undue reservation.

## Ethics statement

This study was reviewed and approved by The Ethics Committee of the Third Affiliated Hospital of Shenzhen University. The patients/participants provided their written informed consent to participate in this study.

## Author contributions

MP was a major contributor in designing the study and writing the manuscript. XD, XZ, and MY helped with the design of the study. AT, XY and FZ collected the data. DX, RJ and TO analysed and interpreted the data. WL designed the study and revised the article. All authors contributed to the article and approved the submitted version.

## Funding

The study was supported by Shenzhen Science and Technology Plan Project Key Technical Tackling Project (JSGG20200225151806035), Shenzhen Science and Technology Plan General Project (JCYJ20210324131203011).

## Conflict of interest

The authors declare that the research was conducted in the absence of any commercial or financial relationships that could be construed as a potential conflict of interest.

## Publisher’s note

All claims expressed in this article are solely those of the authors and do not necessarily represent those of their affiliated organizations, or those of the publisher, the editors and the reviewers. Any product that may be evaluated in this article, or claim that may be made by its manufacturer, is not guaranteed or endorsed by the publisher.

## Abbreviations

AUC, area under the curve; BMI, body mass index; CE, conformational epitope; COVID-19, coronavirus disease 2019; ISC2V, Sinovac’s inactivated SARS-CoV-2 vaccine; FDR, false discovery rate; FG, original data foreground; NAb, neutralizing antibody; PBSTP, phosphate buffered saline with 0.05% Tween20 and 0.1% ProClin^TM^; RBD, receptor binding domain; ROC, receiver operating characteristic; S, spike; SARS-COV-2, severe acute respiratory syndrome coronavirus 2; SDP, significantly differentiated peptide; VIABR, vaccine-induced antibody-binding region.
